# Labradorins with Antibacterial Activity Produced by *Pseudomonas* sp.

**DOI:** 10.3390/molecules22071072

**Published:** 2017-06-27

**Authors:** Anders Broberg, Joakim Bjerketorp, Pierre Andersson, Christer Sahlberg, Jolanta J. Levenfors

**Affiliations:** 1 Department of Molecular Sciences, Uppsala BioCenter, the Swedish University of Agricultural Sciences, P.O. Box 7015, SE-750 07 Uppsala, Sweden; joakim.bjerketorp@slu.se (J.B.); mailapierre@gmail.com (P.A.); jolanta.levenfors@slu.se (J.J.L.); 2 Medivir AB, P.O. Box 1086, SE-141 22 Huddinge, Sweden; christer.sahlberg@gmail.com

**Keywords:** antibiotics, secondary metabolites, labradorin, oxazolylindole, antibiotic resistant bacteria, *Pseudomonas*, *Staphylococcus aureus*, *Acinetobacter baumannii*

## Abstract

The urgent need for new antibacterial drugs has led to renewed interest in microorganisms, which historically have been the main source of previously discovered antibiotics. The present study describes the discovery of two new antibacterial oxazolylindole type alkaloids, labradorins 5 (**1**) and 6 (**2**), which were isolated and characterized from two isolates of *Pseudomonas* sp., along with four previously known tryptophane derived alkaloids. The structures of **1** and **2** were determined by NMR spectroscopy and MS, and confirmed by synthesis. During bioassay-guided isolation using several human bacterial pathogens, **1** and **2** displayed activity towards *Staphylococcus aureus* and *Acinetobacter baumannii*. The minimal inhibitory concentrations (MIC) of compounds **1** and **2** against *S. aureus* were 12 μg·mL^−1^ and 50 μg·mL^−1^, respectively, whereas the MICs against *A. baumannii* were >50 μg·mL^−1^. The CC_50_ values of compound **1** towards a liver cell line (HEP-G2) and a T-cell line (MT4) were 30 μg·mL^−1^ and 20 μg·mL^−1^, respectively, and for compound **2** were >100 μg·mL^−1^ and 20 μg·mL^−1^, respectively. Due to the limited potency of compounds **1** and **2**, along with their toxicity, the compounds do not warrant further development towards new antibiotics.

## 1. Introduction

Antibiotic resistant bacteria have become a major threat to global health and there is an urgent need for new antibacterial drugs [1]. The majority of all antibiotics used today and in the past are either microbial natural products or compounds derived from natural products [2]. Throughout evolution, bacteria and fungi have developed a rich variety of antimicrobial compounds to combat other microorganisms in competition for limited nutrients. Because of the diversity of antimicrobial compounds produced by bacteria and fungi, natural product based antibiotics still dominate over the purely synthetic ones when comparing antibacterial drugs developed and introduced since 1981 [3].

The last decades have seen a significant improvement of chromatographic and spectroscopic techniques for isolation and characterization of minute amounts of secondary metabolites as well as tools for rapid de-replication to avoid known compounds. The improvements include the development of high-performance liquid chromatography (HPLC) and, more recently, ultra-high performance liquid chromatography (UHPLC) for efficient isolation/separation of natural products in complex mixtures, and high-resolution mass spectrometry (HRMS) techniques and cryogenic nuclear magnetic resonance (NMR) probes for structure determination of small amounts of natural products [4,5]. Additionally, the hyphenation of chromatographic and MS techniques, e.g., UHPLC-HRMS, has been shown to be very efficient in profiling of extracts and for de-replication purposes [4,5]. In parallel, genomic techniques, such as microbial genome mining [6,7], have been developed that enable selection of microbial isolates talented in the production of secondary metabolites, and more recently, new methods for isolating previously uncultivable bacteria [8]. Screening microorganisms for production of secondary metabolites with antibacterial properties has once again become a promising approach for the discovery of new antibacterial compounds.

During the last years, we have screened bacterial and fungal isolates for the discovery of novel antibacterial secondary metabolites. The present study describes the isolation and characterization of two new antibacterial oxazolylindole type alkaloids (**1** and **2**) from isolates of *Pseudomonas* sp., along with four other, previously known, tryptophane derived alkaloids.

## 2. Results and Discussion

### 2.1. Isolation of Compounds

**Figure 1:**
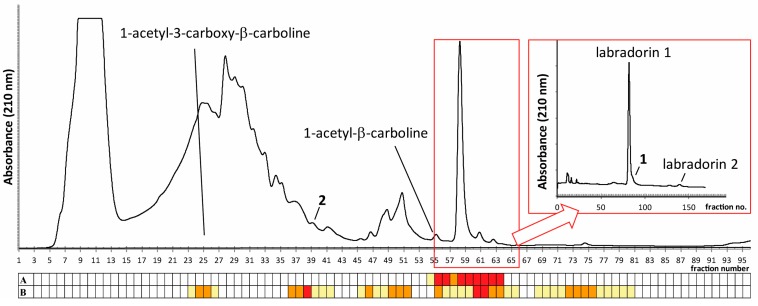
Gradient reversed-phase HPLC fractionation of culture extracts of *Pseudomonas* sp. Ki341. Colored boxes below the chromatogram indicate activity towards A: *Acinetobacter baumannii* and B: *Staphylococcus aureus* (red: full inhibition of bacterial growth; white: no inhibition of bacterial growth; yellow and orange: intermediate inhibition). The inset shows isocratic fractionation of fractions 55–65, with indications of the peaks of labradorins 1 and 2, and compound **1**.

**Figure 2:**
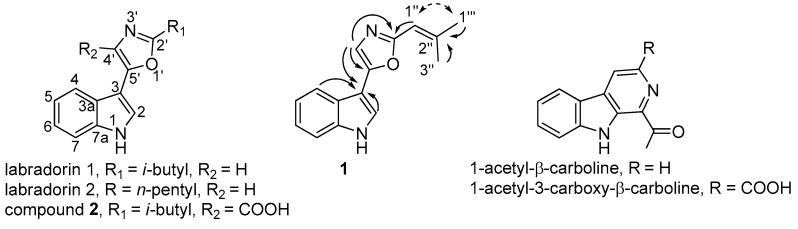
Structures of labradorins 1 and 2, compounds **1** and **2**, and 1-acetyl-β-carboline and 1-acetyl-3-carboxy-β-carboline. Key correlations from heteronuclear multiple bond correlation (HMBC, solid lines) and rotating-frame nuclear Overhauser effect spectroscopy (ROESY, dashed lines) experiments are included in the structure of compound **1**.

Bioassay-guided reversed-phase HPLC fractionation of spent culture broths from *Pseudomonas* sp. isolate Ki341 [9,10], using a quick gradient of acetonitrile in water, resulted in several fractions with activities against the opportunistic pathogens *Acinetobacter baumannii* and *Staphylococcus aureus* (Figure 1). LC-HRMS analysis of selected fractions, followed by a database search, suggested labradorins 1 and 2 to be major compounds in the extract [11] (Figure 2), and also suggested that several other related compounds may be present in the sample. Following culture up-scaling, the gradient separation with associated bioassays was repeated (Figure 1) and the fractions suspected to contain labradorins 1 and 2 were pooled and subjected to isocratic HPLC (45% MeCN, 0.2% formic acid). The respective fractions containing labradorins 1 and 2, as indicated by LC-HRMS, were pooled, dried and analyzed by NMR, and the resulting NMR data was in good agreement with published data for the two compounds [11].

### 2.2. Identification of Compound ***1***

The bioassays from the isocratic HPLC step indicated no or low activity for labradorin 1 and labradorin 2 towards *S. aureus*, just as previously described for the compounds [11]. However, fractions on the shoulder of the labradorin 1 peak showed activity towards *S. aureus*, and LC-HRMS analysis of these fractions indicated a labradorin 1 analogue (**1**) with one further unsaturation or ring in the structure. Subsequent one-dimensional ^1^H-NMR analysis clearly showed that compound **1** was indeed similar to labradorin 1—the signals from H-2, H-4, H-5, H-6 and H-7 of the indole moiety and the H-4′ signal of the oxazole part were present at chemical shifts similar to the corresponding signals in labradorin 1 (Table 1, [11], Figure S1). Nonetheless, the signals of the isobutyl group were not present; instead, a signal from a sp^2^ CH was present along with two signals from methyl groups—both singlet signals at chemical shifts indicating the methyl groups to be linked to a sp^2^ carbon. Thus, the ^1^H-NMR data suggested a structure for compound **1** similar to labradorin 1, the only difference being a double bond between C-1′′ and C-2′′ (Figure 2), all in line with the HRMS data, which suggested the molecular formula C_15_H_14_N_2_O for the compound. The tentative structure of compound **1** was verified by extensive two-dimensional NMR spectroscopy, as indicated by key correlations from heteronuclear multiple bond correlation (HMBC) and rotating-frame nuclear Overhauser effect spectroscopy (ROESY) experiments (Figure 2).

**Figure 3:**
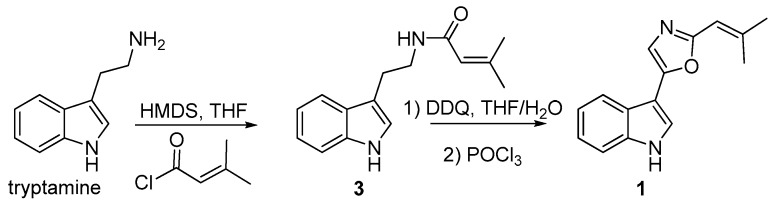
Synthesis of compound **1** [11,12].

Due to the presence of large amounts of labradorin 1 in the fractions eluting only slightly before the fractions with compound **1**, it was difficult to isolate sufficiently pure compound **1** for evaluation of biological activities. Therefore, compound **1** was synthesized as summarized in Figure 3, similar to previous syntheses of labradorins [11,12]. The synthesized compound was subsequently shown by NMR, LC-HRMS and LC-HRMSMS to be identical to the natural **1**, which finally confirmed the identity of compound **1**, which was given the name labradorin 5.

### 2.3. Identification of Compound ***2***

Another suspected labradorin type compound (**2**) eluted earlier than labradorin 1 in the gradient separation above (Figure 1). LC-HRMS data suggested the formula C_16_H_16_N_2_O_3_ for **2**, i.e., one extra carbon and two extra oxygen atoms compared to labradorin 1. Labradorin 1 has previously been shown to be biosynthesized from the amino acids tryptophane and leucine [12]. Thus, compound **2** was hypothesized to be an analogue of labradorin 1, still having the tryptophane carboxylic acid group linked to C-4′. Compound **2** eluted in a more complex part of the chromatogram than compound **1**, and indeed it was found to be difficult to obtain sufficient amounts of this compound from *Pseudomonas* sp. isolate Ki341, for successful NMR analysis. These difficulties were overcome by the use of another isolate of *Pseudomonas* sp. termed Mb36: 2 [13], which proved to produce more of compound **2** than Ki341. Bioassay-guided HPLC fractionation of culture broths of isolate Mb36: 2 yielded, after two steps of chromatography, sufficient amounts of compound **2** for NMR analysis. The ^1^H-NMR data of **2** (Table 1, Figure S2) was similar to labradorin 1; the major difference was the absence of the H-4′ signal, which clearly indicated that the extra carbon and the two oxygen atoms were positioned at C-4′ as a carboxylic acid group. To verify the suggested structure and to obtain pure material for biological evaluation, the proposed compound **2** was purchased (custom synthesis by BHM, China). Subsequent comparison by NMR, LC-HRMS and LC-HRMSMS confirmed the proposed structure of compound **2**, which was given the name labradorin 6.

### 2.4. Identification of Known β-Carbolines

Further active fractions from the gradient reversed-phase HPLC fractionation of culture extracts of *Pseudomonas* sp. isolate Ki341, were found to contain another type of indole based alkaloids, the known β-carbolines 1-acetyl-β-carboline and 1-acetyl-3-carboxy-β-carboline (Figure 1 and Figure 2). This was determined by LC-HRMS analysis of relevant fractions and subsequent NMR analysis after isocratic fractionation of the indicated fractions. The NMR data was in good agreement with literature values for both compounds [14,15,16]. The 1-Acetyl-3-carboxy-β-carboline has previously only been found in plants [15,17], and this is the first report on the production of this compound by a bacterium. Conversely, 1-acetyl-β-carboline has previously been isolated from several types of organisms, including plants [14,18], a fungus [19], a sponge [20], and several bacteria e.g., [21].

### 2.5. Biological Activities of Compounds ***1*** and ***2***

The minimal inhibitory concentrations (MIC) of compounds **1** and **2** towards *S. aureus* were determined to be 12 μg·mL^−1^ and 50 μg·mL^−1^, respectively. The MIC values of both compounds towards *A. baumannii* were >50 μg·mL^−1^. The very similar compound labradorin 1 has previously been reported to be inactive against *S. aureus* at concentrations up to 64 μg·mL^−1^ [11]. Notably, compound **1** displayed more than 5-fold higher activity towards *S. aureus* compared to labradorin 1. This difference was observed already during purification of compound **1**, as no activity towards *S. aureus* was detected in fractions containing large amounts of labradorin 1, while neighboring fractions with lower concentrations of **1** showed activity. The only difference between the compounds is the 2-methylprop-1-enyl group in **1**, instead of an isobutyl group in labradorin 1, which gives the two compounds slightly different shapes and possibly also different chemical reactivities. The cytotoxicity (CC_50_) of compound **1** towards HEP-G2 (liver cell line) and MT4 (T-cell line) cells was 30 μg·mL^−1^ and 20 μg·mL^−1^, respectively. The corresponding CC_50_ values for compound **2** were >100 μg·mL^−1^ and 20 μg·mL^−1^, respectively. In comparison, labradorin 1 showed GI_50_ values (concentrations giving 50% growth inhibition) for a selection of human cell lines between 3.5 and 9.8 μg·mL^−1^ [11]. The observed MIC value for compound **1** towards *S. aureus* was not low enough to merit further evaluation as a potential antibacterial drug candidate. In addition, the CC_50_ values were only 2–3-fold higher than the MIC value, which gives a therapeutic window that is too narrow for the application of the compound as an antibacterial drug.

## 3. Materials and Methods

### 3.1. General Experimental Procedures

UV-spectra were recorded on a Hitachi U-2001 spectrophotometer in MeOH at room temperature. Preparative HPLC was run on a Gilson 306/306 pump system (Gilson Inc., Middleton, WI, USA) with a Gilson 119 UV/VIS detector monitoring at 210 nm. Fractions were collected with a Gilson 204 fraction collector and collection was done in polypropylene 2.2 mL square well plates (VWR, Radnor, PA, USA). For the mobile phase, MeCN of HPLC gradient grade (Sigma-Aldrich, St. Louis, MO, USA) and deionized filtered water (Millipore, Billerica, MA, USA) were used. LC-HRMS was performed on an Agilent 1100 HPLC (Agilent, Palo Alto, CA, USA) connected to a maXis Impact Q-TOF MS (Bruker Daltonic GmbH., Bremen, Germany). ^1^H and ^13^C-NMR data was acquired on a Bruker Avance III 600 MHz NMR spectrometer (Bruker Biospin GmBH, Rheinstetten, Germany) equipped with a 5-mm cryo-probe (^1^H, ^13^C, ^15^N, ^31^P). Standard pulse sequences supplied by Bruker were used for determination of ^1^H and ^13^C frequencies and connectivities. For complete structure elucidation, 1D ^1^H, COSY, TOCSY, NOESY, DEPT-HSQC and HMBC were applied. Chemical shifts were determined relative to internal chloroform (δ_C_ 77.23; δ_H_ 7.27).

### 3.2. Isolate Origin, Identity and Maintenance

The psychrotrophic *Pseudomonas* sp. isolates Ki341 [9,10] and MB36:2 [13] have been described previously. *Pseudomonas* sp. isolate Ki341 originates from the roots of wild dicotyledonous plant collected in the vicinity of Kiruna (Sweden). *Pseudomonas* sp. isolate MB36:2 was separated from a culture of Pink-Pigmented Facultative Methylotrophic (PPFM) bacterium MB36 originating from clover (*Trifolium repens*/*T. pratence*), which was collected near Uppsala, Sweden. The isolates were assigned to the genus *Pseudomonas*, RNA group I, by means of morphological physiological and biochemical tests, and 16S rRNA sequencing. Sequencing of the full-length 16S rRNA genes assigned all isolates to the “*Pseudomonas chlororaphis* group”, and these isolates were most similar (but not identical) to the species of *P. mandelii* and/or *P. frederiksbergensis*. The isolates were maintained as deep-frozen (−70 °C) stock cultures. When appropriate, cultures were transferred to Vegetable Peptone Broth agar plates (VPA, 10 g Vegetable Peptone Broth (Oxoid Ltd., Basingstoke, UK), 15 g Bacto Agar (Difco Ltd., Detroit, MI, USA) in 1 L deionised water).

Isolates of *Escherichia coli* LMG15862, *Acinetobacter baumannii* LMG1041T, *Enterobacter cloacae* LMG2783T, *Klebsiella pneumoniae* LMG20218, *Pseudomonas aeruginosa* LMG6395, *Staphylococcus aureus* LMG15975 were purchased from Belgian Co-ordinated Collections of Micro-organisms, Gent, Belgium) and maintained as advised by the Collection. The isolate of *Candida albicans* (Robin) Brekhout was kindly provided by the Laboratory of Clinical Microbiology, Centre of Laboratory Medicin, University Hospital, Uppsala, Sweden. The isolate of *Aspergillus fumigatus* Fres. originates from the Department of Microbiology, SLU, Uppsala, Sweden. Cells of *C. albicans* and spores of *A. fumigatus* were produced as previously described [22]. All cell/spore stocks were stored at −70 °C.

### 3.3. Culture Conditions and Metabolite Sampling

During primary screening for antimicrobial activity, the cultures of isolates (150 mL in 500 mL Erlenmeyer flasks) were grown in two substrates: half strength Vegetable Peptone Broth (VPB; 15 g VPB (Oxoid Ltd.) in 1000 mL deionised H_2_O) and in modified Mineral Medium (MM) for *Pseudomonas* [23,24]. The 150 mL-VPB cultures were initiated either by transferring 100 μL of deep-frozen isolate stock per 150 mL VPB or by transferring a loop (10 μL) of 24–48-h-old cell material grown on VPA plates (VBP + 1.5% agar). The MM cultures were started by transferring 1.5 mL of 24-h-old VPB cultures per 150 mL MM. For production of selected active metabolites, isolates were cultured in half strength VPB (300 mL in 1000 mL Erlenmeyer flasks). The cultures were always incubated on a rotary shaker (130 rpm) for 96–120 h at 20 °C in the darkness. In order to collect extracellular metabolites, sterile nylon bags with a polymeric resin Amberlite XAD 16 (Sigma-Aldrich; approximately 5 g per bag for 150 mL and 10 g per bag for 300 mL cultures) were submerged in actively growing cultures 16 to 24 h after inoculation.

### 3.4. Sample Work-Up and Isolation Procedures

One Amberlite XAD-16 bag from a 150 mL culture of *Pseudomonas* sp. Ki341 was washed with deionized water to remove bacterial cells and culture resins. The bag was subsequently extracted with 2 × 20 mL MeOH and 20 mL MeCN. The combined extracts were dried at 30–35 °C under nitrogen gas flow. The dried material was subjected to gradient preparative reversed phase HPLC (21.2 × 100 mm, 5 μm, Hypersil Gold, Thermo Scientific, Waltham, MA, USA) using a gradient of MeCN in water (10–95% MeCN in 10 min, followed by 10 min at 95% MeCN, at 10 mL min^−1^). Fractions were bioassayed and analyzed by LC-HRMS as described below. For isolation of larger amounts of compounds **1**, a total of ten Amberlite XAD-16 bags from 10 × 150 mL cultures of isolate Ki341were washed as described above and extracted with, in total, 2 × 200 mL MeOH and 2 × 200 mL MeCN. Gradient HPLC fractionation was done as above, and fractions 55–65, containing labradorins 1 and 2, along with compound **1**, as indicated by bioassays and LC-HRMS (as below), were pooled, dried and subjected to isocratic reversed phase HPLC (45% MeCN, 0.2% formic acid, 10 mL min^−1^, column as above). Larger amounts of compound **2** from isolate Mb36:2 were isolated analogously, but fractions 34–38 from the gradient fractionation (as above) were subjected to isocratic separation at 40% MeCN (0.2% formic acid).

### 3.5. *In Vitro* Bioassay

During primary screening and during isolation of compounds **1** and **2**, fractions with antibacterial/antifungal activity were identified using the “Microtiter plate assay 2” protocol described previously [25], which is based on inhibition of cell growth or spore germination in microtiter plates. *E. coli* LMG15862, *A. baumannii* LMG1041T, *E. cloacae* LMG2783T, *K. pneumoniae* LMG20218, *P. aeruginosa* LMG6395, *S. aureus* LMG15975, *C. albicans* and *A. fumigatus* were used as indicator organisms. Briefly, aliquots of the HPLC fractions were transferred into 96-well microtiter plates and the solvent was evaporated in a fume-hood overnight. Cell suspensions (all bacterial isolates and *C. albicans*) or spore suspensions (*A. fumigatus*), 100 μL at a concentration of 10^4^ cells/spores mL^−1^ in growth medium (VBP for bacteria and Malt Extract (ME, Difco Ltd., Detroit, MI, USA) for fungi) were added to the wells and incubated at 37 °C in darkness for 16 to 24 h. Inhibition of bacterial/fungal growth was estimated visually according to the following scale: 3 = full growth inhibition, 2 = intermediate growth inhibition, 1 = weak growth inhibition, 0 = no growth inhibition. Cell/spore suspensions without HPLC fractions were used as positive controls, and sterile medium as negative control. Following isocratic HPLC separations, only *S. aureus* and *A. baumannii* were used as activity indicators.

### 3.6. Analysis by LC-HRMS

Selected fractions were analyzed by LC-HRMS on a reversed phase HPLC column (3.0 × 50 mm, 2.6 μm, Accucore RP-MS, Thermo Scientific, Waltham, MA, USA) using a gradient of MeCN in water, both with 0.2% formic acid (10–95% MeCN in 3 min, 95% MeCN for 4 min, at 0.8 mL min^−1^). The MS was operated in positive mode with scanning of *m*/*z* 50–1500, and the mass spectra were calibrated against sodium formate clusters.

### 3.7. MIC Determination

The MIC determinations of compounds **1** and **2** were done by the broth micro-dilution method in 96-well microtiter plates with the addition of *S. aureus* and *A. baumannii* as test organisms. The test media were either AM3 Broth (Difco Ltd., Detroit, MI, USA) mixed with phosphate buffer pH 7.0 in 1:4 ratio or AM3 Broth mixed with PBS in 1:1 ratio. Cell concentration of tested pathogens (*A. baumannii* and *S. aureus*) was adjusted to between 1 and 5 × 10^5^ cells mL^−1^. The numbers of estimated viable cells were confirmed directly after each MIC test by plating diluted subsamples of the respective pathogen on VPA plates, incubating these overnight at 37 °C and then counting colonies. In order to establish MIC, appropriate volumes of solutions of test compounds (100, 10, 1 and if needed 0.1 μg mL^−1^) dissolved in MeOH were dispensed into wells of 96-well microtiter plates and the solvent was evaporated. MIC of the test compounds was tested in a concentration range between 0.05 and 50 μg mL^−1^. Cells of pathogen suspended in test medium were distributed to the wells and the plates were incubated for 16 to 20 h at 37 °C. Wells containing only medium were used as negative controls while wells with the pathogen in medium without test compound were used as positive controls. The MIC was defined as the lowest concentration of each compound with no visible pathogen growth. All MIC tests were performed in triplicate and repeated at least twice. Cell growth was monitored following 16 to 20 h incubation at 37 °C in darkness.

### 3.8. Toxicity Determination

MT4 (T-cell line, a gift from Prof. Yamamoto, Yamaguchi University, Yamaguchi, Japan) and HEP-G2 (human liver cell line, ATCC, VA) cell lines were passaged into 96-well microplates (2 × 10^4^ cells per well) followed by the addition of the test substances the next day, in duplicate samples. The number of viable cells was assessed after 6 days by using a soluble formazan (XTT) assay [26] and the compound INX-189 was used as positive control [27].

### 3.9. Synthesis of Compound ***1***

To a stirred solution of tryptamine (0.80 g, 5 mmol) and hexamethyldisilazane (2.09 mL, 10 mmol) in THF (20 mL), under nitrogen and at room temperature, 3-methylbut-2-enoyl chloride (0.67 mL, 6 mmol) was added over a 5 min period. The reaction mixture was then stirred for 48 h and acidified with 0.1 M HCl. The reaction mixture was extracted twice with ethyl acetate and the combined organic phases washed with saturated NaHCO_3_ and brine. The organic phase was dried over Na_2_SO_4_, concentrated and purified on a silica gel column eluting with ethyl acetate/hexanes 1:1 to yield 1.18 g (97.4%) of *N*-(2-(1*H*-indol-3-yl)-ethyl)-3-methylbut-2-enamide (**3**). ESIMS *m*/*z* 243.1 [M + H^+^]. ^1^H-NMR (400 MHz, DMSO-*d*_6_) δ 1.77 (s, 3H), 2.08 (s, 3H), 2.81 (t, 2H), 3.34 (m, 2H), 5.63 (s, 1H), 6.97 (t, 1H), 7.06 (t, 1H), 7.13 (d, 1H), 7.33 (d, 1H), 7.53 (d, 1H), 7.82 (t, 1H), 10.78 (s, 1H).

Compound **3** (100 mg, 0.41 mmol) was dissolved in THF (9 mL), and water (1 mL) and DDQ (187 mg, 0.83 mmol) was added. The reaction mixture was stirred at room temperature for a period of 2 h. Then, the reaction mixture was concentrated and 0.1 M NaOH (5 mL) was added and the mixture was vigorously stirred overnight. The intermediate product was collected by filtration and 63 mg of a yellow solid was obtained (ESIMS *m*/*z* 257.1 [M + H]^+^). This product was dissolved in POCl_3_ (1.5 mL) and heated at 105 °C overnight. The mixture was allowed to cool down and poured into a mixture of ice water (8 mL) and methanol (2 mL) and extracted 3 times with dichloromethane. The combined organic phases were dried over Na_2_SO_4_, concentrated and purified on a silica gel column eluting with ethyl acetate/hexanes 1:4 to yield 4 mg (4%) of 5-(1*H*-indol-3-yl)-2-(2-methylprop-1-en-1-yl)oxazole, (labradorin 5, **1**).

Labradorin 5 (**1**): Off-white powder; UV (MeOH) λ_max_ (log^1^ ε): 224 (4.19), 320 (4.08); ^1^H-NMR (CDCl_3_, 600 MHz), see Table 1; ^13^C-NMR (CDCl_3_, 150 MHz), see Table 1; ESI-QTOFMS *m*/*z* 239.1182 [M + H]^+^ (calcd for C_15_H_15_N_2_O^+^ 239.1179).

Labradorin 6 (**2**): Off-white powder; UV (MeOH) λ_max_ (log^10^ ε): 224 (4.14), 275 (3.80), 317 (3.89); ^1^H-NMR (CDCl_3_, 600 MHz), see Table 1; ^13^C-NMR (CDCl_3_, 150 MHz), see Table 1; ESI-QTOFMS *m*/*z* 285.1237 [M + H]^+^ (calcd for C_16_H_17_N_2_O_3_^+^ 285.1234).

## 4. Conclusions

Labradorins 5 (**1**) and 6 (**2**) were isolated from *Pseudomonas* sp. based on their activity against *S. aureus* and *A. baumannii*. The MIC values subsequently measured for **1** and **2** towards *S. aureus* were 12 μg mL^−1^ and 50 μg mL^−1^ respectively, and for *A. baumannii* the value was >50 μg mL^−1^ for both compounds. The CC_50_ values for **1** and **2** towards human cell lines were of similar size to the MIC values. In conclusion, the MIC and CC_50_ values for **1** and **2** do not support further evaluation of the compounds as antibacterial drug candidates.

## Figures and Tables

**Table 1:** ^1^H- and ^13^C-NMR data for compounds **1** and **2** (CDCl_3_, 30 °C, 600/150 MHz).

	Compound 1			Compound 2		
Pos.	δ_C_	δ_H_	Mult. (Hz)	δ_C_	δ_H_	Mult. (Hz)
1	-	8.38	br. s	-	8.61	br. s
2	122.3	7.56	d (2.3)	130.1	8.95	d (2.8)
3	105.7	-	-	104.0	-	-
3a	124.3	-	-	124.9	-	-
4	120.3	7.86	d (7.9)	121.4	8.18	d (7.5)
5	121.6	7.27	t (7.9)	122.1	7.30	t (7.5)
6	123.7	7.31	t (7.9)	123.6	7.33	t (7.5)
7	112.1	7.46	d (7.9)	112.0	7.48	d (7.5)
7a	136.4	-	-	136.0	-	-
1′	-	-	-	-	-	-
2′	159.6	-	-	160.2	-	-
3′	-	-	-	-	-	-
4′	119.4	7.33	s	122.2	-	-
5′	146.7	-	-	154.2	-	-
1′′	111.3	6.31	br. s	37.1	2.80	d (7.3)
2′′	147.5	-	-	27.8	2.31	m
3′′	21.3	2.32	s	22.6	1.10	d (6.6)
1′′′	28.1	2.04	s	22.6	1.10	d (6.6)
COOH	-	-		162.2	n.d. ^a^	-

^a^ Not detected.

## Supplementary Materials

Supplementary materials are available online. Figure S1: 1D ^1^H-NMR spectrum of labradorin 5 (**1**), Figure S2: 1D ^1^H-NMR spectrum of labradorin 6 (**2**), Figure S3: UV spectra of labradorins 5 (**1**) and 6 (**2**).
