# Supplementary material for: Labradorins with Antibacterial Activity Produced by Pseudomonas sp
Source: Molecules. 2017 Jun 27;22(7):1072. doi: 10.3390/molecules22071072 (PMC6151975; doi:10.3390/molecules22071072)
Supplement: Supplementary file 1 [file molecules-22-01072-s001.pdf]

molecules

MDPI

Supplemental Materials

Antibacterial Labradorins from *Pseudomonas* sp.

Anders Broberg [1,*], Joakim Bjerketorp [1], Pierre Andersson [1], Christer Sahlberg [2], and Jolanta Levenfors [1]

[1] Department of Molecular Sciences, Uppsala BioCenter, the Swedish University of Agricultural Sciences, P.O. Box 7015, SE-750 07 Uppsala, Sweden
[2] Medivir AB, P.O. Box 1086, SE-141 22 Huddinge, Sweden
* Correspondence: anders.broberg@slu.se; Tel.: +46-18-672217

Figure S1: 1D ^{1}H NMR spectrum of labradorin 5 (**1**) in $CDCl_3$ (600 MHz, 30°C).

Figure S2: 1D ^{1}H NMR spectrum of labradorin 6 (**2**) in $CDCl_3$ (600 MHz, 30°C).

Figure S3: UV spectra of labradorins 5 (**1**) and 6 (**2**) in MeOH (22°C).

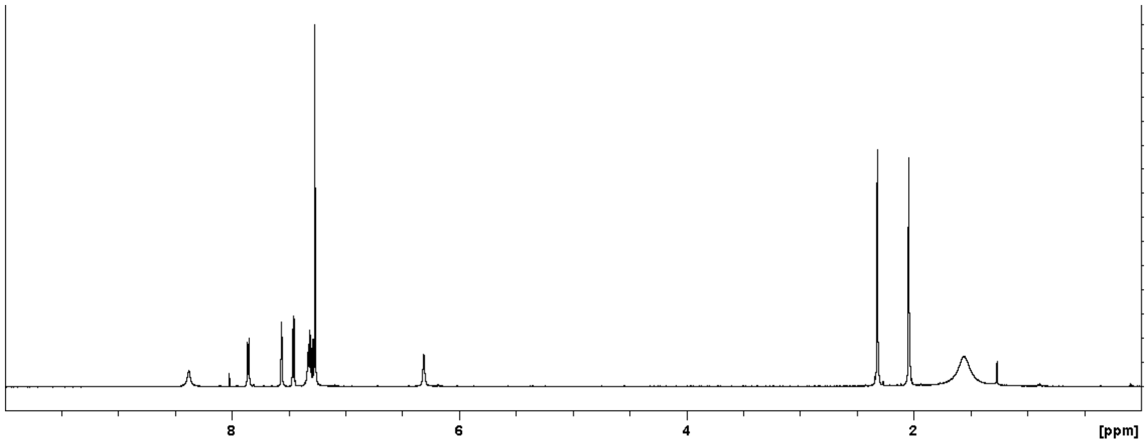

Figure S1: 1D ^{1}H NMR spectrum of labradorin 5 (**1**) in $CDCl_3$ (600 MHz, 30°C).

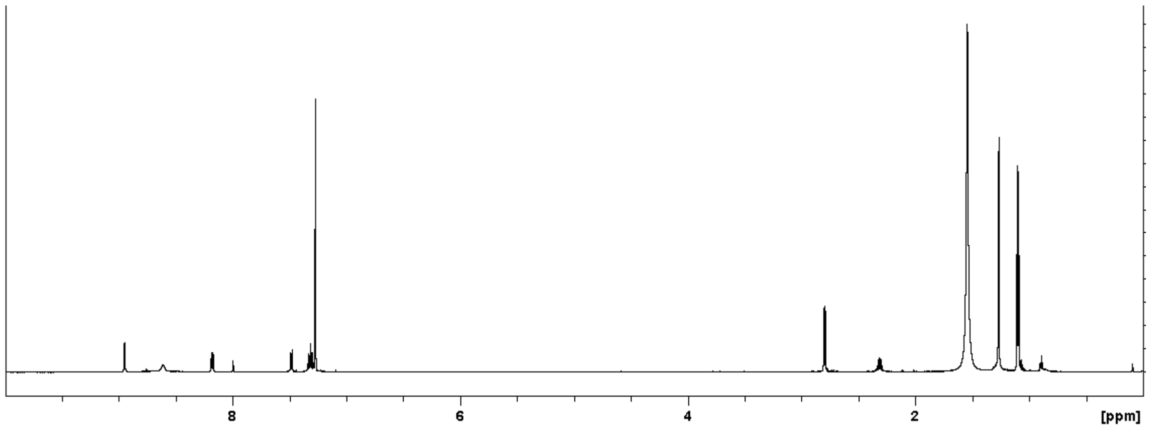

Figure S2: 1D ^{1}H NMR spectrum of labradorin 6 (**2**) in $CDCl_3$ (600 MHz, 30°C).

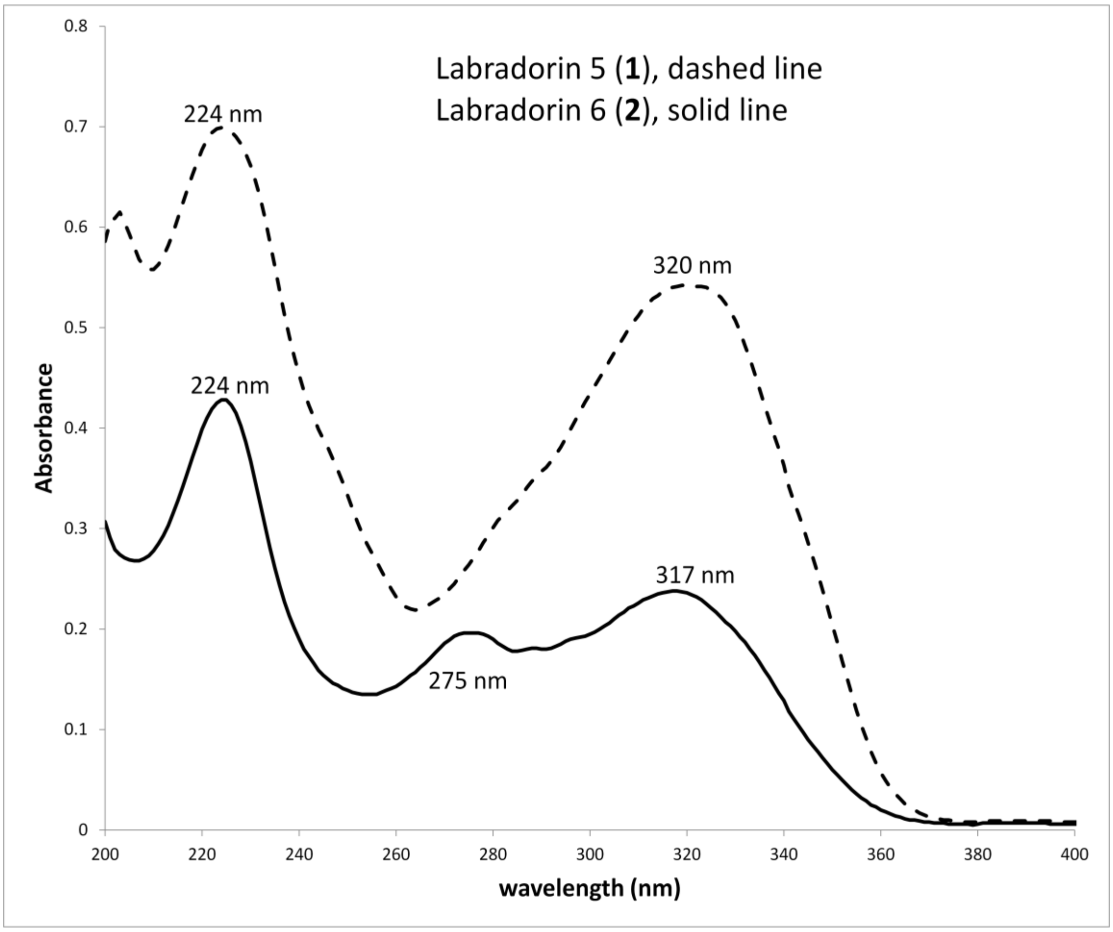

Figure S3: UV-spectra of labradorins 5 (**1**) and 6 (**2**) recorded in MeOH at 22°C.
